# Editorial: The applications of new multi-locus GWAS methodologies in the genetic dissection of complex traits, volume II

**DOI:** 10.3389/fpls.2023.1340767

**Published:** 2023-12-01

**Authors:** Yuan-Ming Zhang, Zhenyu Jia, Jim M. Dunwell

**Affiliations:** ^1^ College of Plant Science and Technology, Huazhong Agricultural University, Wuhan, China; ^2^ Department of Botany and Plant Sciences, University of California, Riverside, Riverside, CA, United States; ^3^ School of Agriculture, Policy and Development, University of Reading, Reading, United Kingdom

**Keywords:** genome-wide association study, mixed linear model, multi-locus model, mrMLM, omics big dataset

Since the inception of multi-locus genome-wide association study (GWAS) methodologies (Segura et al., 2012; Liu et al., 2016; Wang et al., 2016), they have been widely applied to dissect the genetics of complex traits (Zhang et al., 2019). Recently, new methodologies such as 3VmrMLM (Li et al., 2022) have been established, resulting in numerous applications. Therefore, it is imperative to consolidate insights into the advantages and potential limitations of using these advanced multi-locus methods.

## 1 Multi-locus genome-wide association study methods

The evolution of GWAS methods can be divided into three phases: the initial phase of single-marker analysis (Risch and Merikangas, 1996), followed by the emergence of mixed-model-based methods (Zhang et al., 2005; Yu et al., 2006; Kang et al., 2008; Kang et al., 2010; Zhang et al., 2010; Segura et al., 2012; Zhou and Stephens, 2012; Liu et al., 2016), and more recently the integration of mixed models with machine learning methods (Wang et al., 2016; Wen et al., 2018; Li et al., 2022). Currently, rapid single-locus genome-wide scans and multi-locus two-step methods are widely used. However, advocates are leaning towards mixed model plus machine learning methods, such as 3VmrMLM (Li et al., 2022), as they comprehensively consider all effects while controlling for all polygenic backgrounds.

In most methods, the marker genotypes QQ, Qq and qq are typically coded as 2, 1 and 0 respectively, indicating their breeding values in a random mating population. In this context, the parameter to be estimated is the allele substitution effect (*α*), controlling for the *α*-based polygenic background, as in FASTmrEMMA. Locus identification becomes difficult as *α* approaches zero. Meanwhile, the detection of dominant effects proves challenging due to the small coefficient resulting from similar frequencies of two alleles at a locus, and significant differences in the frequencies of two alleles are equivalent to the presence of a rare allele. When these genotypes are coded as 1, 0 and -1, the estimated effect becomes the additive effect, as seen in methods such as mrMLM. This method is particularly applicable when the majority of marker genotypes are homozygous, as observed in crops such as rice, wheat and soybean. However, the assumption of random mating often does not fit well. Therefore, these situations can lead to reduced power and contribute to missing heritability. To solve these challenges, a recommended approach is to include all effects in a mixed model while controlling for all polygenic backgrounds, as demonstrated in methods such as 3VmrMLM (Li et al., 2022).

When analyzing real data, the inflation factor or quantile-quantile plot serves as a common metric to assess method performance. This is important for single-marker genome-wide scanning methods, as opposed to the multi-locus two-step mrMLM and 3VmrMLM methods. The latter methods use a more relaxed *P*-value threshold during the genome-wide scan, aiming to select potentially associated markers rather than to identify significant loci. Given the complementary nature of these methods, it is often advisable to employ multiple methods when analyzing a single trait (Zhang et al., 2019). This increases the probability of identifying more significant/suggested loci. A method is good for analyzing the trait when it mines the most known and candidate genes around these loci, in which the candidate genes should be supported by strong evidence. When presenting the results, emphasis can be placed on the highlighted loci containing known and candidate genes in the Manhattan plot.

## 2 The applications of new multi-locus GWAS methodologies in the genetic dissection of complex traits

Disease resistance is a key trait affecting crop yield. Three studies on this topic identified 54 resistance quantitative trait nucleotides (QTNs), while another study compared selection methods that simultaneously improve yield and resistance. Shu et al. used six multi-locus methods to identify 13 QTNs associated with maize resistance to southern corn rust (SCR). Validation included post-GWAS case-control sampling and allele/haplotype analysis. Candidate genes were mined using transcriptomic annotation analysis and confirmed using tissue-specific and stress-induced transcriptomic analysis. Allele/haplotype effects, resistance and susceptibility of each QTN and QTN-QTN combination in breeding were estimated. These authors advocated a diverse panel of well-designed breeding lines, rich in SNP markers, as a more effective approach for the discovery of small effect and broad SCR resistance loci. Channale et al. identified fourteen chickpea accessions resistant to *Pratylenchus thornei* and 24 resistance QTNs using six multi-locus methods, while six candidate genes were identified around these QTNs under biotic and abiotic stresses, although differential expression and functional analyses were not performed. Subsequently, Nandudu et al. performed univariate and multivariate GWAS for cassava brown streak disease (CBSD) severity using GEMMA. Univariate GWAS identified five QTNs and multivariate GWAS identified 17 QTNs. Gene ontology analysis mined trait-related candidate genes. In addition, Mediterranean corn borer resistance studies have shown a strong negative correlation between yield and resistance. To determine the effectiveness of genomic selection over phenotypic selection in improving both traits, Gesteiro et al. compared different selection programmes. Genomic selection proved to be the most successful method for improving yield, although phenotypic or genotypic selection for yield may be more effective for improving both traits simultaneously.

To address the problem of over-application of nitrogen fertilizer and to improve nitrogen use efficiency (NUE), Liao et al. performed GWAS for eleven traits in 419 rice landraces, using 208,993 SNPs and the MLM, mrMLM and 3VmrMLM methods. This investigation led to the identification of key QTNs associated with NUE. Eight known genes and 75 candidate genes were identified around these QTNs, and seven candidate genes were further confirmed by RT-qPCR, including *LOC_Os10g33210* and *LOC_Os05g51690*. The results provide valuable genetic resources for molecular breeding of rice cultivars with improved NUE.

## 3 Future perspectives

Large-scale gene mining is a crucial aspect of future research efforts. On the one hand, the loci identified offer cost-effective opportunities for genomic selection in crops. On the other hand, the wealth of candidate genes around these loci can be explored using multi-omics analysis. In the multi-omics era, a wide range of data, databases, platforms and techniques are becoming available. The integration of genetic loci with multi-omics information is emerging as an inevitable trend that will shape the future of research and exploration in this field.

## Author contributions

Y-MZ: Writing – original draft, Writing – review & editing. ZJ: Writing – review & editing. JD: Writing – review & editing.
